# Modifiable and non-modifiable risk factors of dementia on midlife cerebral small vessel disease in cognitively healthy middle-aged adults: the PREVENT-Dementia study

**DOI:** 10.1186/s13195-022-01095-4

**Published:** 2022-10-12

**Authors:** Audrey Low, Maria A. Prats-Sedano, Elizabeth McKiernan, Stephen F. Carter, James D. Stefaniak, Stefania Nannoni, Li Su, Maria-Eleni Dounavi, Graciela Muniz-Terrera, Karen Ritchie, Brian Lawlor, Lorina Naci, Paresh Malhotra, Clare Mackay, Ivan Koychev, Craig W. Ritchie, Hugh S. Markus, John T. O’Brien

**Affiliations:** 1 Department of Psychiatry, School of Clinical Medicine, University of Cambridge, Box 189, Level E4 Cambridge Biomedical Campus, Cambridge, Cambridgeshire CB2 0SP UK; 2 Department of Clinical Neurosciences, University of Cambridge, Cambridge, UK; 3 Department of Neuroscience, University of Sheffield, Sheffield, UK; 4 Centre for Dementia Prevention, University of Edinburgh, Edinburgh, UK; 5 INSERM, Montpellier, France; 6 Institute of Neuroscience, Trinity College Dublin, University of Dublin, Dublin, Ireland; 7 Division of Brain Science, Imperial College Healthcare NHS Trust, London, UK; 8 Department of Psychiatry, Oxford University, Oxford, UK; 9 Cambridgeshire and Peterborough NHS Foundation Trust, Cambridge, UK

**Keywords:** Alzheimer’s disease, Cerebral small vessel disease, Modifiable risk factors, APOE4, Lifestyle, Prevention

## Abstract

**Background:**

Considerable overlap exists between the risk factors of dementia and cerebral small vessel disease (SVD). However, studies remain limited to older cohorts wherein pathologies of both dementia (e.g. amyloid) and SVD (e.g. white matter hyperintensities) already co-exist. In younger asymptomatic adults, we investigated differential associations and interactions of modifiable and non-modifiable inherited risk factors of (future) late-life dementia to (present-day) mid-life SVD.

**Methods:**

Cognitively healthy middle-aged adults (aged 40–59; mean 51.2 years) underwent 3T MRI (*n* = 630) as part of the PREVENT-Dementia study. To assess SVD, we quantified white matter hyperintensities, enlarged perivascular spaces, microbleeds, lacunes, and computed composite scores of SVD burden and subtypes of hypertensive arteriopathy and cerebral amyloid angiopathy (CAA). Non-modifiable (inherited) risk factors were APOE4 status and parental family history of dementia. Modifiable risk factors were derived from the 2020 Lancet Commission on dementia prevention (early/midlife: education, hypertension, obesity, alcohol, hearing impairment, head injuries). Confirmatory factor analysis (CFA) was used to evaluate the latent variables of SVD and risk factors. Structural equation modelling (SEM) of the full structural assessed associations of SVD with risk factors and APOE4*risk interaction.

**Results:**

In SEM, the latent variable of global SVD related to the latent variable of modifiable midlife risk SVD (*β* = 0.80, *p* = .009) but not non-modifiable inherited risk factors of APOE4 or family history of dementia. Interaction analysis demonstrated that the effect of modifiable risk on SVD was amplified in APOE4 non-carriers (*β* = − 0.31, *p* = .009), rather than carriers. These associations and interaction effects were observed in relation to the SVD subtype of hypertensive arteriopathy, rather than CAA. Sensitivity analyses using separate general linear models validated SEM results.

**Conclusions:**

Established modifiable risk factors of future (late-life) dementia related to present-day (mid-life) SVD, suggesting that early lifestyle modifications could potentially reduce rates of vascular cognitive impairment attributed to SVD, a major ‘silent’ contributor to global dementia cases. This association was amplified in APOE4 non-carriers, suggesting that lifestyle modifications could be effective even in those with genetic predisposition to dementia.

**Supplementary Information:**

The online version contains supplementary material available at 10.1186/s13195-022-01095-4.

## Introduction

Cerebral small vessel disease (SVD) occurs frequently in older adults, and is increasingly implicated in cognitive impairment and dementia, contributing to a significant proportion of dementia cases worldwide [1]. In fact, post-mortem studies report concomitant cerebrovascular disease in large proportions of Alzheimer’s disease (AD) cases, whereby mixed AD + vascular pathology commonly exceeds “pure” AD cases [2]. In addition to the overlapping pathologies of AD and SVD, there exists a considerable body of evidence on their overlapping risk factors.

Importantly, SVD can occur many years ahead of symptom onset, with converging evidence from neuroimaging and post-mortem studies—in both sporadic and familial AD—implicating SVD in *early* AD pathogenesis [3, 4]. Despite this, existing studies on the overlapping risk factors of AD and SVD have been restricted to elderly or symptomatic cohorts, wherein pathological markers of AD and concomitant vascular pathologies are already co-existing [2], thereby limiting one’s ability to disentangle the differential contributions to distinct disease pathologies.

To overcome these limitations, investigations must be conducted earlier in the lifespan, during the early preclinical phases of incipient dementia. Therefore, we aimed to examine whether established risk factors of future late-life dementia confer risk of SVD much earlier in life, at midlife, years before cognitive symptoms of dementia are expected. This extension of the literature to midlife cohorts is necessary to elucidate early pathological processes and improve early detection and intervention. This is especially important in light of the emerging evidence of differential effects of risk factors on brain structure across the lifespan, whereby risk factors demonstrate stronger predictive value when measured at midlife, relative to late-life [5–9].

Classification of risk factors can help discriminate between varying sources of risk, and thereby determine the degree of agency that individuals have in controlling their own risk of dementia, and guide precision medicine and early prevention strategies. Therefore, in our cohort of healthy middle-aged adults, we considered two forms of risk: modifiable and non-modifiable risk. Non-modifiable (inherited) risk was defined by the possession of the APOE4 allele and parental family history of dementia (FH), both of which are established risk factors of AD [10, 11]. Modifiable risk was based on the early and midlife risk factors identified in the *2020 Lancet Commission* on dementia prevention, intervention, and care [12].

We hypothesised that midlife SVD burden would relate to both modifiable and non-modifiable risk factors of dementia. Furthermore, we expected regional differences in the associations between SVD and modifiable risk factors, whereby modifiable risk would relate more closely to SVD distribution indicative of hypertensive arteriopathy (lesions in deep subcortical regions, e.g. basal ganglia), rather than the SVD subtype of cerebral amyloid angiopathy (CAA-SVD; e.g. lobar microbleeds, lobar lacunes), given that hypertensive arteriopathy commonly involves injury to deep perforating arteries susceptible to hypertension-related alterations, while the latter relates to the deposition of Aβ within vessel walls [1, 13–15]. Finally, we examined the interaction between modifiable risk factors and APOE4 on midlife SVD—considering that the combination of modifiable risk factors (e.g. hypertension) and APOE4 relates to increased risk of SVD in elderly participants [16, 17], we hypothesised that the effect of modifiable risk on SVD burden would be similarly amplified in middle-aged APOE4 carriers.

## Methods

### Participants

The protocol of the PREVENT-Dementia study has been described in detail previously [18, 19]. Briefly, participants were recruited from five sites across the UK and Ireland (West London, Edinburgh, Cambridge, Oxford, Dublin). The PREVENT-Dementia study was designed to identify the earliest predictors of dementia. Therefore, participants had to be cognitively healthy (absence of dementia and other neurological conditions) middle-aged adults, aged 40 to 59, making it one of the youngest cohorts in dementia research at present. The sample was enriched for dementia risk through the oversampling of individuals with parental family history of dementia (FH), with the aim of achieving an equal split of those with and without FH, resulting in a corresponding increase in the proportion of APOE4 carriers.

**Fig. 1:**
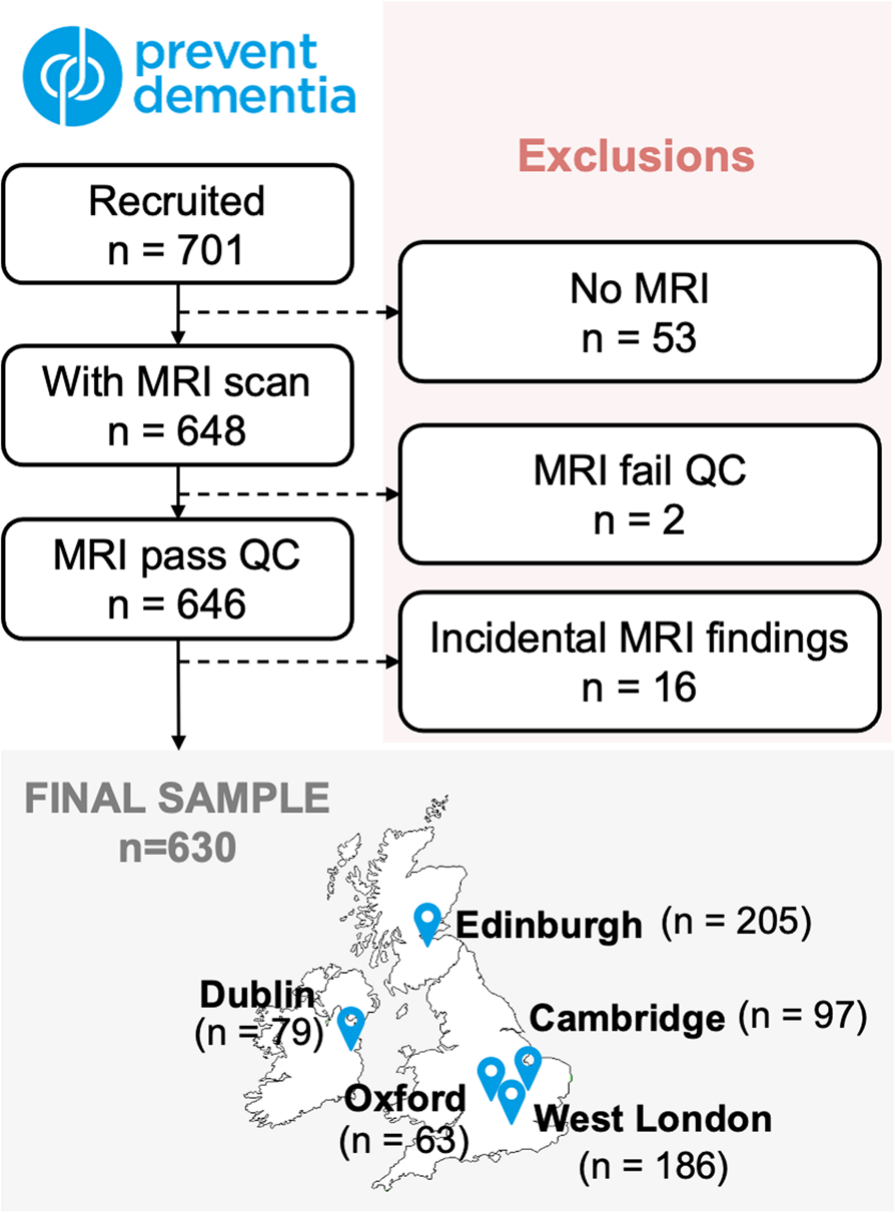
Participant selection flowchart

Of the 701 participants recruited, 648 underwent MRI scanning. Scans were obtained between 2014 and 2021. All scans were visually inspected—two participants were excluded from analysis due to poor image quality (e.g. image artefacts), and 16 were excluded due to incidental findings (e.g. meningioma, tumour resection), resulting in a final sample size of 630. Details on each step and the breakdown of participants per study site can be found in Fig. 1.

### MRI acquisition parameters

Three-dimensional T1-weighted (T1w) MPRAGE parameters were as follows: 160 slices, repetition time (TR) = 2300 ms, echo time (TE) = 2.98 ms, flip angle = 9°, voxel size = 1 × 1 × 1 mm^3^. T2-weighted (T2w) parameters were as follows: 32 slices, TR = 1500 ms, TE = 80 ms, flip angle = 150°, voxel size = 0.69 × 0.69 × 4 mm^3^. Fluid-attenuated inversion recovery (FLAIR) parameters were as follows: 27 slices, TR = 9000 ms, TE = 94 ms, flip angle = 150°, voxel size = 0.43 × 0.43 × 4 mm^3^. Susceptibility weighted imaging (SWI) parameters were as follows: 72 slices, TR = 28 ms, TE = 20 ms, flip angle = 15°, voxel size = 0.72 × 0.72 × 1.2 mm^3^.

### Quantification of SVD

#### Semi-quantitative measurements

**Fig. 2:**
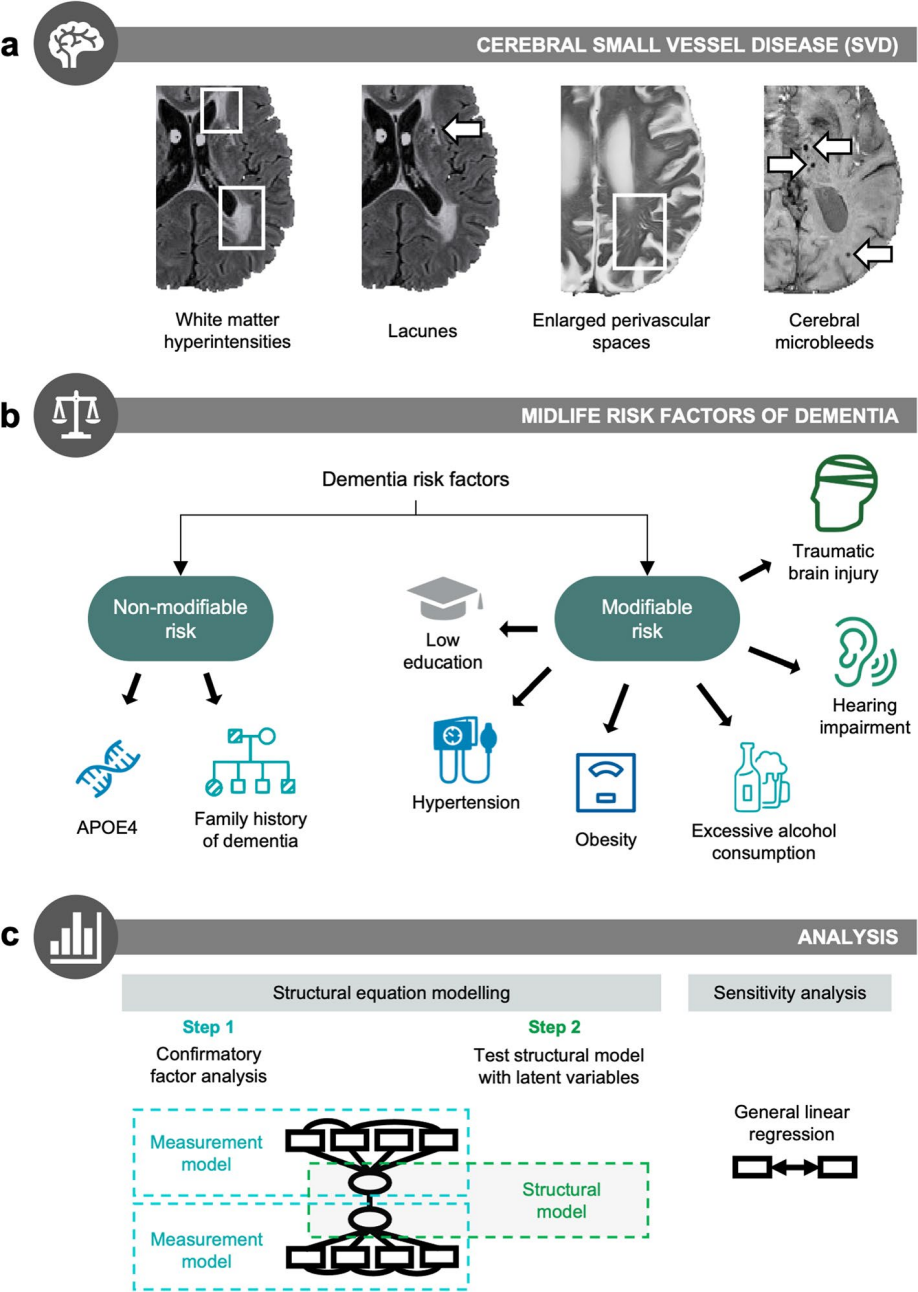
Overview of methodology. **a** Cerebral small vessel disease (SVD) was quantified on 3T MRI using four imaging markers: white matter hyperintensities, lacunes, enlarged perivascular spaces, and cerebral microbleeds. **b** Midlife risk factors were classified into two categories: non-modifiable (inherited) risk vs. modifiable risk factors. Non-modifiable risk factors were APOE4 and parental family history of dementia. Modifiable risk factors were based on the risk factors identified in the 2020 Lancet Commission report for dementia prevention [12] (early to mid-life risk factors). **c** Structural equation modelling was performed to assess associations between the latent variables of modifiable risk and SVD. The first stage tests the measurement model using confirmatory factor analysis. If measurement models achieve good fit, the second stage tests the full structural model. As a form of sensitivity analysis, results were replicated using general linear regression models to determine the robustness of results across different methods. Detailed statistical procedures can be found under the “Methods” section

SVD quantification protocols have been described in detail previously [20] (Fig. 2a). WMH were visually rated on FLAIR MRI according to the Fazekas scale for the computation of composite SVD scores [21]. Periventricular and deep WMH were rated separately: periventricular (0 = absent; 1 = “caps” or pencil-thin lining; 2 = smooth “halo”; 3 = irregular periventricular signal extending into the deep WM) and deep (0 = absent; 1 = punctate foci; 2 = beginning confluence; 3 = large confluent areas). EPVS were rated on T2w MRI using a validated rating scale [22] and cross-checked with T1w MRI. EPVS were assessed separately in the basal ganglia and centrum semiovale to account for different underlying pathologies. EPVS scores ranged from 0 to 4 according to lesion count: 0 (none), 1 (1–10), 2 (11–20), 3 (21–40), 4 (> 40). CMB were identified on SWI following the Microbleed Anatomical Rating Scale (MARS) [23]. Suspected CMB were cross-validated on T1w and T2w scans to exclude CMB ‘mimics’. In instances of uncertainty, CMB were labelled as ‘possible CMB’—this includes situations whereby CMB cannot be distinguished from vascular flow voids. Such cases of ‘possible CMB’ were excluded from analysis, and only ‘definite CMB’ were analysed. Suspected CMB were cross-validated on T1w and T2w scans to exclude CMB ‘mimics’. SWI scans were not available for 7 participants and data from 17 participants had to be excluded to due to low image quality. Lacunes were identified using T1w, T2w, and FLAIR scans, following the STRIVE guidelines [24]. Lacunes and CMB were classified according to location as deep (e.g. basal ganglia, thalamus) or lobar (e.g. centrum semiovale) lesions [13, 23]. Lacunes and CMB data were dichotomised separately as ‘present’ (at least one lesion) or ‘absent’ (no lesions).

Each SVD marker was rated by a single rater, and 20% of scans were rated by a second rater. The subset of 20% was derived from random sampling by a third party of all participants stratified by study site. Raters were blinded to all clinical information, and inter-rater reliability (Cohen’s kappa) were as follows: CMB: 0.74, lacunes: 0.92, EPVS: 0.90 in centrum semiovale, 0.85 in basal ganglia, WMH: 0.74 for periventricular, and 0.89 for deep. All discrepancies and uncertain cases were discussed, and consensus was reached amongst raters.

#### Quantitative measure of WMH volume

WMH lesion maps were obtained from FLAIR images using an automated script on SPM12 (http://www.fil.ion.ucl.ac.uk/spm/); details described elsewhere [25]. Briefly, SPM12 was used to perform segmentation of T1w images into gray matter (GM), white matter (WM), and cerebrospinal fluid (CSF), based on prior probability maps. Using the GM and WM maps, a brain mask was created and used to perform removal of non-brain matter from the FLAIR images. Initial WMH maps were then obtained using threshold-based segmentation at a threshold of 1.2 times the median pixel intensity of the whole brain, i.e. lesions with pixel intensity more than 1.2 times the median intensity were included in the WMH lesion map. All lesion maps were reviewed by a single experienced rater blinded to all clinical information. Lesion maps obtained from the automated segmentation procedure were used as starting points for manual WMH delineation. WMH were segmented into periventricular or deep WMH based on threshold distance from ventricles, as previously described [25].

Binary ventricular masks underwent four iterations of morphological dilation in MNI space. These dilated ventricular masks were then transformed to individual subject space to define the boundary between periventricular and deep WMH. Due to the transformation, these were variable distances accounting for individual brain size, and were approximately 10 mm, in line with published recommendations [26]. WMH volumes were normalised by total intracranial volume (TIV) to account for individual differences in head size ((WMH/TIV) * 100%) and underwent cube root transformation due to right-tailed skewness.

#### Composite SVD scores

Composite scores of global SVD burden were computed according to Staals and colleagues [27], while the two SVD subtypes of CAA and hypertensive arteriopathy were formulated according to distinctions made in the literature [13–15, 28, 29], following a similar scoring system for comparability. For CAA, one point was awarded for each criterion met: (1) one or more lobar lacunes, (2) one or more lobar CMB, (3) EPVS score in centrum semiovale ≥ 2, and (4) periventricular WMH Fazekas = 3 and/or deep WMH Fazekas ≥ 2. Similarly, for hypertensive arteriopathy: (1) one or more deep lacunes, (2) one or more deep CMB, (3) EPVS score in basal ganglia ≥ 2, and (4) deep WMH Fazekas ≥ 2 (Supplementary Table 1).

Our composite score of CAA-SVD does not include all radiological markers of CAA (e.g. intracranial haemorrhage, cortical superficial siderosis). For the sake of accessibility, our simplified scoring system of CAA-SVD (and hypertensive arteriopathy) was modelled after an existing rating scale by Staals and colleagues [26], such that researchers with the data to compute this composite SVD burden score should be able to compute the two composite scores of SVD subtypes, i.e. hypertensive arteriopathy and CAA-SVD, assuming that some minimal regional information (e.g. deep/lobar) is captured during visual rating. In other words, some degree of exactness was sacrificed for the sake of (1) accessibility and (2) comparability between the three composite scores.

### Genotyping

TaqMan genotyping on QuantStudio12K Flex was used to establish APOE variants. Genomic DNA was isolated from whole blood and genotyping was performed in 384 well plates, using the TaqMan polymerase chain reaction (PCR)-based method.

### Modifiable risk factors

All participants underwent comprehensive clinical assessment. Risk factors were based on the *2020 Lancet Commission* on dementia prevention [12] (Fig. 2b). Given the age profile of the PREVENT-Dementia cohort (age 40–59), we focused on early and midlife modifiable risk factors: low education, hearing impairment, TBI, hypertension, high alcohol intake, and obesity [30]. Apart from hearing loss and TBI which were dichotomous variables, continuous measures of the remaining risk factors were loaded onto the latent risk variable, i.e. years of education, systolic blood pressure, waist-to-hip ratio, and units of alcohol per week. The rationale for using latent modelling is described in the following section (see the “Structural equation modelling” section).

For the purposes of interaction analysis and post hoc sensitivity analysis, a composite midlife risk score was derived by dichotomizing each risk factor using the following cut-offs, based on published guidelines where available: low education (< 13 years), hearing loss (self-reported), traumatic brain injury (≥ 1 event of head injury resulting in loss of consciousness), high alcohol intake (> 21 units/week) [12], hypertension (systolic BP ≥ 140 or diastolic BP ≥ 90; National Institute for Health and Care Excellence (NICE) guidelines, NG136, 2019), and obesity (waist-to-hip ratio ≥ 0.78 for females or ≥ 0.87 for males [31]) [12]. The composite risk score was then derived by assigning 1 point to each risk factor, except for hypertension and obesity which were assigned 2 points each, given their stronger loadings onto the latent risk construct in CFA (see the “Modifiable midlife risk factors relate to SVD burden” section).

### Statistical analysis

#### Structural equation modelling

Given the multifactorial nature of both SVD and dementia risk, SEM is preferred over standard general linear regressions due to its ability to evaluate complex multivariate models and test associations between *global* dimensions (i.e. latent variables; not measured in dataset), which are defined by multiple individual variables (i.e. observed/measured variables; directly measured in dataset). Furthermore, the use of latent variables reduces the extent of measurement error which could artificially attenuate associations between measured (observed) variables in univariate analysis. These considerations are especially crucial for our study, given that SVD burden is estimated by multiple non-independent measures of SVD, just as modifiable risk can be assessed as a composite of multiple risk variables.

#### Confirmatory factor analysis of measurement model

Before entering the latent variables into a full structural model, the latent variables themselves are evaluated. This is known as confirmatory factor analysis (CFA), which is typically the first step of SEM analysis involving latent variables and serves to evaluate the associations between latent variables (e.g. SVD) and their indicators (e.g. WMH, lacunes)—this is known as a *measurement model* which is used to verify the measurement quality of latent variables that are included in the *full structural model* (Fig. 2c). The latent variable of SVD burden was estimated by the four imaging markers of SVD, i.e. total WMH volume, EPVS in the basal ganglia, presence of microbleeds, and presence of lacunes [27]. The latent variable of modifiable risk was estimated from the six modifiable risk factors in early-life and mid-life identified in the *2020 Lancet Commission* report [12].

#### Structural equation modelling of full structural model

To assess the associations between the latent variables of SVD and risk, full structural models were fitted separately for *midlife* risk and *lifetime* risk, with path direction specified as *Risk ➔ SVD*, and accounting for sex (0 = female, 1 = male), age (in years), and APOE4 status (0 = APOE4 non-carrier, 1 = APOE4 carrier). Education was not included as a ‘covariate’ since it is accounted for within the latent risk variable. Regression coefficients were assessed, and the following hypothesised paths were removed due to non-significance in separate steps: *APOE4➔SVD*, *APOE4➔RISK*, *SEX➔SVD* (final full structural model under the “Results” section).

#### Interaction analysis within full structural model

To assess the interaction of APOE4 with modifiable risk on SVD, the APOE4*RISK interaction term was added to the SEM model as a predictor of SVD. Given the inability of *lavaan* (R package for SEM) to process interactions with *latent* variables, a measurable (observed) risk variable was required. Therefore, the point-based midlife risk score (see the “Modifiable risk factors” section) was used.

#### Model fit evaluation

Model fit was assessed using the comparative fit index (CFI), root mean square error of approximation (RMSEA), and standardized root mean square residual (SRMR). CFI is an index of ‘good fit’, whereby higher values indicate better model fit—values above or close to 0.95 are considered good fit. The latter two (RMSEA, SRMR) are indices of “bad fit”, whereby lower values are preferred—values below 0.1 are acceptable, while values below 0.05 are considered to indicate good fit [32].

#### Model modification

For each model, modification indices were examined to identify potential sources of misfit that could improve model fit if addressed. Re-specifications were considered when modification indices were large (> 3.84) and implemented if the proposed changes were theoretically sound.

#### Simple regression modelling

To test whether FH and APOE4 related to SVD burden, we used Spearman’s correlation for unadjusted analysis. To adjust for covariates, we independently fitted regression models to each SVD marker—simple linear regression models were fitted to WMH and EPVS, while logistic regression models were fitted to binary variables (presence of CMB/lacunes). In all regression models, we adjusted for sex (0 = female, 1 = male), age (centred at sample mean of 51.2 years), and study site (nominal variable with 5 levels).

To assess the robustness of our findings, we conducted a series of post-hoc sensitivity analyses. In clinical research, sensitivity analysis is performed to assess the extent to which results are affected by changes in methods [33]). This was done by replicating SEM results in general linear regression models, adjusting for sex, age, and study site. Specifically, to test the association between modifiable risk and SVD, the derived composite risk score was fitted to composite SVD scores in simple linear regression models. To examine the APOE4*RISK interaction on SVD observed in our structural model, the interaction term was added to the base model with the same covariates, as earlier described. To further validate our APOE4 interaction results, we fitted independent linear regression models with the same covariates by analysing APOE4 carriers and non-carriers in separate models.

All statistical analyses were conducted using R version 4.0.3 (www.R-project.org). Models were estimated under a missing at random missing data assumption using maximum likelihood estimation. To reduce collinearity, all independent variables in regression models were mean-centred. Correction for multiple comparisons was implemented using false discovery rate (FDR) correction to control for type 1 error for the parameters within the SEM model. SEM analysis was conducted using the *lavaan* package. The *lm* function was used to fit linear regression models, and *glm* was used for logistic regression modelling. Statistical significance was set at *p* < 0.05.

## Results

Sample characteristics are summarised in Table 1. The sample had a larger proportion of females (*n* = 388, 61.6%), a mean age of 51.2 (SD = 5.5), and an average of 16.7 years of education (SD = 3.4). As study recruitment aimed to enrich the sample for AD risk, 53.3% had a family history of dementia, while 38.4% were APOE4 carriers. In terms of modifiable risk factors, participants had an average of 1.0 risk factor each (median = 1); 23.6% were not positive on any risk factor, 33.8% had one risk factor, 27.6% had two risk factors, and 15.1% had three or more risk factors.

**Table 1:** Participant characteristics

***N***		630
**Demographics**		
Sex	% females	61.6%
Age (in years)	Mean (SD)	51.2 (5.5)
Education (in years)	Mean (SD)	16.7 (3.4)
APOE4	% carriers	38.4%
APOE2	% carriers	8.4%
Hypertension	% positive	16.5%
Hyperlipidaemia	% positive	12.1%
Diabetes mellitus	% positive	2.7%
Obese	% positive	26.2%
Excessive alcohol consumption	% positive	14.1%
Hearing impairment	% positive	10.8%
Traumatic brain injury	% positive	35.4%
**Medication**		
Anti-hypertensive medication	% on medication	7.3%
Anti-hyperlipidemic medication	% on medication	5.2%
Anti-diabetic medication	% on medication	6.2%
**SVD markers**		
WMH volume (% of TIV)	Mean (SD)	0.13 (0.16)
Lacunes (present/absent)	% present	10.5%
Cerebral microbleeds (present/absent)	% present	16.8%
Enlarged perivascular spaces (range 0–4)	Mean (SD)	0.94 (0.49)
**Composite SVD scores**		
Global SVD (range 0–4)	Mean (SD)	0.44 (0.72)
CAA (range 0–4)	Mean (SD)	0.49 (0.69)
Hypertensive arteriopathy (range 0–4)	Mean (SD)	0.27 (0.60)

*Abbreviations*: *SD* standard deviation, *SVD* small vessel disease, *WMH* white matter hyperintensities, *TIV* total intracranial volume, *CAA* cerebral amyloid angiopathy. Missing data: Education (*n* = 1), APOE (*n* = 5), hyperlipidaemia (*n* = 18), diabetes mellitus (*n* = 3), WMH volume (*n* = 3), CMB (*n* = 24), composite SVD scores (*n* = 24)

### Non-modifiable (inherited) dementia risk not related to SVD burden

In both the un-adjusted models (Spearman’s correlation) and general linear models adjusting for sex, age, education, and site, neither APOE4 nor FH were related to any SVD markers (Table 2).

**Table 2:** Group differences in cerebral small vessel disease (SVD) burden by family history and APOE4

	Family history				APOE4			
	Unadjusted		Adjusted		Unadjusted		Adjusted	
	Rho	*p* value	*t* value	*p* value	Rho	*p* value	*t* value	*p* value
**WMH**	− 0.05	0.193	− 1.85	0.064	0.04	0.275	1.52	0.129
**EPVS**	0.00	0.914	− 1.45	0.148	0.01	0.885	− 0.10	0.922
**CMB**	0.01	0.856	0.04	0.969	0.04	0.365	1.33	0.184
**Lacunes**	− 0.05	0.176	− 1.50	0.133	− 0.08	0.054	− 1.72	0.085

Unadjusted analyses were conducted using Spearman’s correlation; adjusted analyses were conducted using general linear modelling adjusting for sex, age, education, and study site. *Abbreviations*: *WMH* white matter hyperintensities, *EPVS* enlarged perivascular spaces, *CMB* cerebral microbleeds

### Modifiable midlife risk factors relate to SVD burden

**Fig. 3:**
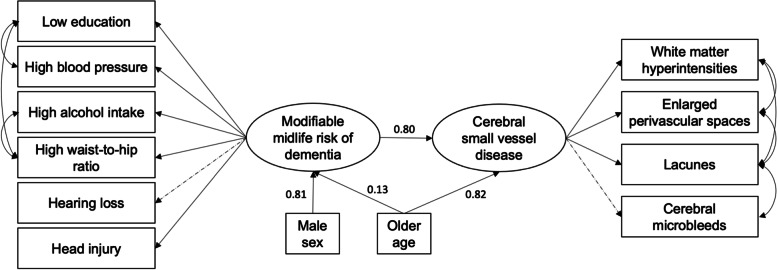
Modifiable midlife risk factors of dementia related to cerebral small vessel disease. Full structural model assessing associations between the latent variables of modifiable midlife dementia risk and cerebral small vessel disease, accounting for the effect of age and sex. Rectangles represent observed variables; ovals represent latent variables. Values represent standardised beta coefficients. Straight lines represent paths, while double-arrowed curved lines represent covariance. Solid lines indicate statistically significant associations; dashed lines indicate non-significant paths

CFA results demonstrated good model fit for both the latent models of modifiable midlife risk (CFI = 0.981, RMSEA = 0.013, SRMR = 0.026) and SVD (CFI = 0.984, RMSEA = 0.035, SRMR = 0.021). Regression coefficients are reported in Supplementary Table 2. In the full structural model, accounting for age and sex (CFI = .904, RMSEA = .045, SRMR = .042), SEM demonstrated a significant association between the latent variables of modifiable risk factors and SVD (*β* = 0.80, *t* = 2.62, *p* = .009) (Fig. 3).

To validate this result, we repeated the association test using general linear models adjusting for sex, age, and study site. The regression model confirmed SEM results, demonstrating a significant association between the composite midlife risk score and composite scores for global SVD (*t* = 3.51, *p* < 0.001) and hypertensive arteriopathy (*t* = 3.53, *p* < 0.001), but not CAA (*t* = 1.51, *p* = .132).

### APOE4 moderates the effect of modifiable midlife risk on SVD

**Fig. 4:**
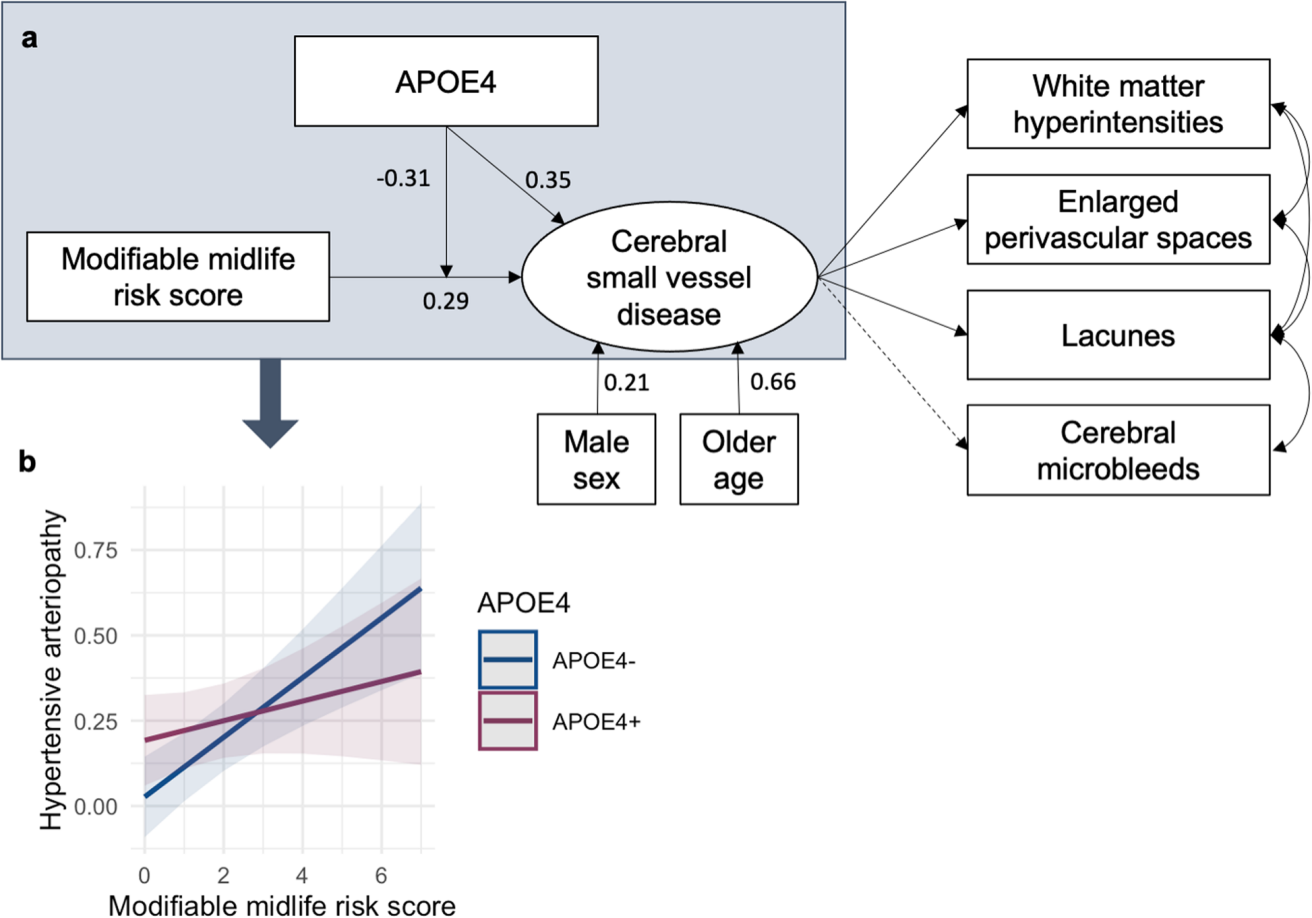
APOE4 moderated associations between modifiable midlife risk and cerebral small vessel disease (SVD). **a** Full structural model assessing associations between the composite score of modifiable midlife risk with the latent SVD variable accounting for age and sex, and the moderating effect of APOE4 on their association. Rectangles represent observed variables; ovals represent latent variables. Values represent standardised beta coefficients. Solid lines indicate statistically significant associations; dashed lines indicate non-significant paths. **b** Interaction plot of marginal effects derived from separate regression analysis fitting the risk*APOE4 interaction term on the composite hypertensive arteriopathy score, adjusting for sex, age, and study site

SEM analysis demonstrated a significant interaction between the composite risk score and APOE4 on the latent SVD variable (*β* = − 0.31, *t* = − 2.62, *p* = .009), such that associations between modifiable risk factors and greater SVD burden were more pronounced in APOE4 non-carriers, relative to APOE4 carriers (Fig. 4). Model fit indices demonstrated excellent fit (CFI = .975, RMSEA = .027, SRMR = .025).

As a form of sensitivity analysis, general linear models were used to confirm findings. Adjusting for sex, age, and site, APOE4 moderated the effect of the composite midlife risk score on the composite score for hypertensive arteriopathy (*t* = − 2.04, *p* = 0.042), but not for global SVD (*t* = − 1.66, *p* = 0.098) or CAA (*t* = − 1.89, *p* = 0.060). These findings were further validated by fitting separate linear regression models in APOE4+ and APOE4- groups separately, which showed significant associations between the modifiable risk score and composite SVD scores in APOE4- (global SVD: *t* = 3.95, *p* < 0.001; CAA: *t* = 2.55, *p* = 0.011, hypertensive arteriopathy: *t* = 3.77, *p* < 0.001) but not APOE4+ (global SVD: *t* = 0.93, *p* = 0.355; CAA: *t* = − 0.40, *p* = 0.691, hypertensive arteriopathy: *t* = 1.05, *p* = 0.296).

## Discussion

In this study, we found that established modifiable risk factors of future late-life dementia was associated with existing SVD burden as early as midlife. Conversely, midlife SVD was not related to non-modifiable (heritable) risk factors like APOE4. Interestingly, associations between modifiable risk and SVD were amplified in APOE4 non-carriers, relative to carriers. Furthermore, these effects were observed specifically in relation to the hypertensive subtype of SVD, but not the CAA-SVD subtype.

We found no significant associations between non-modifiable risk factors (family history of dementia, APOE4) and midlife SVD burden. This is consistent with preliminary results published by our group on a subset of PREVENT-Dementia participants [34, 35] and represents the largest investigation of its kind in healthy middle-aged adults at present. However, contrasting results exist. In a meta-analysis of 42 studies, APOE4 was related to greater SVD burden, even when restricting analyses to the general population [36]—notably, individuals in these studies were considerably older than our middle-aged PREVENT cohort. In a longitudinal study of a subset of PREVENT participants, APOE4 and FH was related to longitudinal progression over 2 years, despite being unrelated to baseline severity, suggesting an inflexion point occurring during midlife, beyond which APOE4 accelerates the rate of SVD progression [35].

SVD burden at midlife was related to the modifiable risk factors of dementia identified in the *2020 Lancet Commission* on dementia prevention. This may suggest that the potentially reduceable risk of dementia resulting from lifestyle changes may be attributed (at least partially) to cerebrovascular health as early as midlife. SVD is linked to various pathological processes such as endothelial dysfunction, neuroinflammation, and blood brain barrier dysfunction [37, 38]. Although the temporal ordering of these processes remains uncertain, the antecedence of SVD early in one’s life course may fast-forward the timeline of this cascade of pathological processes and perpetuate the vicious cycle of neurovascular dysfunction in the lead up to dementia [39]. To that end, further investigations are warranted to analyse SVD in relation to hallmark features of AD to elucidate the role of SVD to dementia across the lifespan.

The full structural model revealed further relevant insights. While the hypothesised model specified paths involving sex, age, and APOE4 status as predictors of both the latent variables of modifiable risk and SVD, non-significant paths were removed, specifically the two APOE4 paths and the *SEX➔SVD* path. Examination of path coefficients (Fig. 3) showed that male sex was a significantly stronger predictor of modifiable risk than age, which may be attributed to men having a higher frequency of certain risk factors, e.g. head injuries and excessive alcohol consumption. Secondly, the removal of the *SEX➔SVD* path was notable as it could imply that sex differences in SVD may be partially attributed to men’s greater lifestyle risk, although further replication is warranted. Finally, while the SEM model provided some information on how risk factors covary (e.g. waist-to-hip ratio covaried with low education and high alcohol intake), future studies could be conducted to examine how risk factors cluster.

Somewhat surprisingly, the detrimental effects of modifiable risk on SVD burden were amplified in APOE4 non-carriers, as opposed to carriers. While seemingly counterintuitive, this new finding corresponds with the emerging body of evidence that APOE4 may be selectively protective in midlife, up to a certain age, beyond which the protective effects wane and turn detrimental instead [40–42]. This is consistent with the concept of antagonistic pleiotropy, which posits that certain genes or alleles may impact fitness differently at different stages in life [43]. Evidence for APOE4 as a case of antagonistic pleiotropy include benefits in early life (e.g. enhanced fertility, lower perinatal and infant mortality) and detrimental effects in later life (e.g. dementia, cardiovascular disease) as the forces of natural selection diminish with age [40, 41]. This phenomenon has been observed in relation to lipid buffering, docosahexaenoic acid (DHA; omega-3 fatty acid) uptake, and innate immunity [42, 44], which may contribute to the seemingly protective role of APOE4 on SVD observed in our sample of healthy middle-aged adults.

Differential results were observed in relation to the two different SVD subtypes. Specifically, associations between modifiable risk and SVD were restricted to the hypertensive arteriopathy subtype of SVD, as opposed to CAA-SVD. This differential association could stem from the distinct aetiologies between the two subtypes. CAA-SVD is characterised by the accumulation of ﻿amyloid-β in vessel walls, while hypertensive arteriopathy is underpinned by vascular influences like blood pressure and blood brain barrier dysfunction. Hypertensive arteriopathy is characterised by vascular alterations in deep subcortical regions (e.g. basal ganglia) which are supplied by deep perforating arteries that are vulnerable to structural alterations caused by hypertension and other vascular risk factors. Of note, CAA-SVD was unrelated to both modifiable and non-modifiable (inherited) risk at midlife. While investigations in elderly cohorts report a similar absence of association with vascular risk factors, APOE4 has been linked to greater CAA burden in older age [45]. Taken together with the absence of midlife associations in our present study, the evidence suggests that the adverse effect of APOE4 on CAA may only emerge in older age.

The operationalisation of dementia risk using SEM has advantages in statistical modelling, although its use in clinical practice is less practical. However, replication of our results using a derived risk score (1 point per risk factor, except hypertension and obesity which count for 2 points each based on CFA results) demonstrate the potential utility of modifiable dementia risk score for clinical assessments, although formal validation studies will be required.

Our findings have significant implications on both dementia prevention strategies (at the individual level, in clinical care, and in public healthcare policy making) as well as future research directions. Central to our study’s aim was the focus on early disease processes in middle-age, and the differentiation of non-modifiable vs. modifiable risk. This delineation of the different forms of risk contributing to SVD in cognitively healthy middle-aged adults could help build better predictive models of incipient dementia, identify new therapeutic pathways, and develop personalised treatment plans. Furthermore, our findings could help pave the way for future research to test new and repurposed strategies for ameliorating dementia by elucidating the pathways through which dementia may be delayed or prevented through actionable changes to one’s lifestyle and public health policy aimed at improving vascular health as early as midlife.

### Strengths and limitations

A key strength of this study was the large sample of relatively young and healthy middle-aged adults, which enabled us to investigate early biomarkers to inform early-stage pathological processes. Furthermore, SVD burden was well-characterised in this study, measuring four key imaging markers of SVD (WMH, CMB, lacunes, EPVS) both globally and regionally. Owing to the comprehensive characterisation of SVD burden, we were able to utilise information on lesion distribution to compute composite scores of not only global SVD burden but also of SVD subtypes of hypertensive arteriopathy and CAA-SVD. Another key strength was the use of structural equation modelling, which enabled us to evaluate complex multivariate models and test associations between global dimensions. This was especially pertinent for our study, given the multiple measures of SVD and risk. Relative to similar community samples, the frequency of SVD markers in the PREVENT-Dementia cohort was on the higher end [46], which could be attributed to the oversampling of individuals at higher risk of developing dementia, including carriers of the APOE4 allele (38% study frequency vs. 18% in the UK [47]) which is a risk factor of SVD in older adults, despite group differences not reaching statistical significance in our cohort. Specifically for CMB, higher incidence may be further attributed to improved sensitivity in CMB detection afforded by thin-slice (vs. thick-sliced) 3T (vs. 1.5T) SWI (vs. gradient echo sequences; GRE) MRI scans, which detect up to triple the number of CMB compared to conventional 1.5T GRE [48], as is thought to represent the true frequency of CMB with greater accuracy.

However, limitations should also be considered in the interpretation of results, and as signposts for future investigations. Firstly, the cross-sectional study design precluded us from examining changes over time and from making any causal inferences. Additionally, given that the sample was predominantly white, we were unable to consider cultural and ethnic differences, and applicability of results are not guaranteed to translate to other ethnicities. Furthermore, we had a highly educated sample and a predominance of females, which may further limit the generalisability of findings to the broader population, while the scarcity of lower-educated individuals in our sample could also be linked to issues of range restriction.

Due to the binary classification of CMB and lacune burden (present/absent), our ability to make inferences regarding number of lesions was limited; that being said, majority of CMB/lacune positive cases presented with just a single CMB/lacune, while only a handful had more than one. Additionally, some variables (e.g. hearing impairment, head injuries) relied on self-report, which may introduce information bias. Finally, to expand this line of work, future studies should incorporate measures of cognition and cognitive decline to consider how these factors relate and interact in relation to objective clinical endpoints.

## Conclusions

In summary, our data demonstrated that well-established risk factors of (future) late-life dementia was already related to (present) cerebrovascular disease much earlier, as early as midlife. This contributes to the growing evidence that SVD represents a key factor in early dementia pathogenesis and may account for a sizeable proportion of dementia risk conferred by these well-known risk factors. In particular, we observed that midlife severity of SVD related to modifiable risk factors, but not non-modifiable inherited risk. This suggests that later-life dementia risk could be reduced through the management of early-life cerebrovascular disease, especially hypertensive arteriopathy, and supports the call to incorporate these risk factors into routine clinical assessments for the development of personalised interventions as early as midlife to prevent not only vascular dementia, but also mixed dementia and even AD [12, 49]. Moreover, the reduced risk from lifestyle modifications at midlife appeared to be especially vital for those without the APOE4 allele, which appeared to buffer the effects of modifiable risk factors on SVD burden, at least at midlife. These findings are consistent with the concept of antagonistic pleiotropy, whereby certain genes can confer differential effects at different life stages (e.g. protective in early/mid-life, deleterious in later life), which highlights the need to consider differential pathological trajectories in APOE4 carriers in assessments, prognosis, and intervention strategies, although this warrants further investigation in longitudinal cohorts.

## Supplementary Information

**Additional file 1: Supplementary Table 1.** Scoring of global cerebral small vessel disease (SVD) burden, hypertensive arteriopathy, and cerebral amyloid angiopathy scores. **Supplementary Table 2.** Factor loadings from confirmatory factor analysis of measurement models.

## Data Availability

Data are available upon reasonable request. The data sets generated for this study are available on request to the senior author for non-commercial academic studies but may be subject to some restrictions according to consent and confidentiality.
