# Supplementary material for: Modifiable and non-modifiable risk factors of dementia on midlife cerebral small vessel disease in cognitively healthy middle-aged adults: the PREVENT-Dementia study
Source: Alzheimers Res Ther. 2022 Oct 12;14:154. doi: 10.1186/s13195-022-01095-4 (PMC9554984; doi:10.1186/s13195-022-01095-4)
Supplement: Supplementary file 1 — Additional file 1: Supplementary Table 1. Scoring of global cerebral small vessel disease (SVD) burden, hypertensive arteriopathy, and cerebral amyloid angiopathy scores. Supplementary Table 2. Factor loadings from confirmatory factor analysis of measurement models. [file 13195_2022_1095_MOESM1_ESM.docx]

**Modifiable and non-modifiable risk factors of dementia on midlife cerebral small vessel disease in cognitively healthy middle-aged adults: the PREVENT-Dementia study**

**SUPPLEMENTARY MATERIALS**

**Supplementary Table 1. Scoring of global cerebral small vessel disease (SVD) burden, hypertensive arteriopathy, and cerebral amyloid angiopathy scores.**

|  |  | **Global SVD**  **(Staals et al., 2014)** | **Hypertensive arteriopathy** | **Cerebral amyloid angiopathy** |
| --- | --- | --- | --- | --- |
| One point per criterion met | WMH | Periventricular WMH = 3 and/or  deep WMH = 2 or 3 | Deep WMH = 2 or 3 | Periventricular WMH = 3 and/or  deep WMH = 2 or 3 |
|  | EPVS | EPVS rating in  basal ganglia ≥ 2 | EPVS rating in  basal ganglia ≥ 2 | EPVS rating in  centrum semiovale ≥ 2 |
|  | CMB | CMB present | Deep CMB present | Lobar CMB present |
|  | Lacunes | Lacunes present | Deep lacunes present | Lobar lacunes present |

Supplementary Table 2. Factor loadings from confirmatory factor analysis of measurement models.

| **Latent variable** | **Observed variable** | **β** | **p value** | **FDR-corrected p value** |
| --- | --- | --- | --- | --- |
| Modifiable midlife risk |  |  |  |  |
|  | Low education | 0.30 | - | - |
|  | Hearing loss | 0.08 | .228 | .228 |
|  | Head injury | 0.15 | .039 | .062 |
|  | Alcohol intake | 0.10 | .141 | .161 |
|  | High blood pressure | 0.44 | .001 | .002 |
|  | High waist-to-hip ratio | 0.48 | .001 | .002 |
| Cerebral small vessel disease |  |  |  |  |
|  | White matter hyperintensities | 0.57 | - | - |
|  | Enlarged perivascular spaces | 0.54 | <.001 | <.001 |
|  | Cerebral microbleeds | 0.10 | .100 | .133 |
|  | Lacunes | 0.36 | <.001 | <.001 |
